# The complete genome sequence of the stolbur pathogen “*Candidatus* Phytoplasma solani” from *Pentastiridius leporinus*

**DOI:** 10.1128/mra.00640-24

**Published:** 2024-11-29

**Authors:** Rafael Toth, Bruno Huettel, Omid Eini, Mark Varrelmann, Michael Kube

**Affiliations:** 1 Department of Integrative Infection Biology Crops -Livestock, University of Hohenheim, Stuttgart, Germany; 2 Max Planck Genome-Centre Cologne, Max Planck Institute for Plant Breeding, Köln, Germany; 3 Department of Phytopathology, Institute of Sugar Beet Research, Göttingen, Germany; University of Maryland School of Medicine, Baltimore, Maryland, USA

**Keywords:** syndrome basses richesses, 16SrXII-P phytoplasma, virulence factors

## Abstract

The complete genome of “*Candidatus* Phytoplasma solani” GOE was obtained from the infected vector *Pentastiridius leporinus* by single-molecule real-time sequencing. This 16SrXII-P phytoplasma is associated with the economically important sugar beet disease “syndrome basses richesses.” The genome sequence is an essential resource for diagnosis and understanding pathogen–host interaction.

## ANNOUNCEMENT

*“Candidatus* Phytoplasma solani” strains (Mollicutes) cause various diseases in crops (1). In Germany, the emerging stolbur subgroup 16SrXII-P (2) shares the insect vector *Pentastiridius leporinus* with the gammaproteobacterium “*Candidatus* Arsenophonus phytopathogenicus” (*3). “Ca*. P. solani” is thus not only involved in the “syndrome basses richesses*”* of sugar beet but also causes stolbur in potato (4). These pathogens spread rapidly due to drastically increasing insect vector populations (5). This study provides the genome of the 16SrXII-P strain GOE for assay development, molecular detection, and diversity- and pathogen–host interaction analysis.

For library template preparation, high-molecular weight DNA was extracted from cicada heads from 15 experimentally infected *P. leporinus* individuals (Institute for Sugar Beet Research, Göttingen, Germany) using solid-phase extraction (NucleoBond HMW DNA Kit, Machery-Nagel, Düren, Germany). DNA concentration was measured with a Qubit fluorometer (Thermo Fisher Scientific, Darmstadt, Germany), and for volume reduction Vivacon 500 (Sartorius, Göttingen, Germany) was used. The SMRTbell prep kit 3.0 (PacBio, Menlo Park, USA) was used to generate a high-fidelity library for single-molecule real-time (SMRT) sequencing (6) without additional DNA fragmentation. Small fragments were removed using diluted PacBio AMPure beads (35%) employing the low DNA input protocol (7). Library sequencing was performed on an 8M ZMW SMRT cell using a Sequel II device (PacBio) with Binding Kit 2.0 and Sequencing Kit 2.0 (PacBio) at the Max Planck Genome Centre (Cologne, Germany).

A total of 1,918,824 generated raw reads were processed with SMRTlink suite (PacBio) including adapter trimming and compared against the NCBI nonredundant protein database (downloaded 03.03.2024) via DIAMOND v0.9.30 BLASTX (8). Results were taxonomically binned using MEGAN v6.25.6 (9). A total of 1,568 reads (N_50_ 13.66 kb) were assigned to “*Ca*. Phytoplasma” and assembled with Canu v2.2 (10), estimating a genome size of 800 kb using the pacbio-hifi option.

Overlap prediction was confirmed with BLASTN v2.9.0 (11) and removed with Artemis Genome Browser v18.2.0 (12), setting *dnaA* as the chromosome start. Annotation was done using RAST v2.0 (13), followed by manual curation with BlastKOALA v3.0 (14) and BLASTP v2.9.0 in Artemis. Assembly quality was evaluated with BUSCO v5.5.0 (15) using 151 single-copy orthologs from the Mollicutes class. Taxon affiliation was confirmed via BLASTN on 16SrRNA. Default parameters were used unless stated otherwise.

The circular chromosome of GOE consists of 704,525 bp, with a sequencing coverage of 43.82 x and a GC content of 26.17%. A total of 663 CDS, two rRNA operons, 32 tRNAs, two ncRNAs, and one tmRNA were predicted. BUSCO analysis showed completeness of 95%. No plasmid was identified. 16SrRNA from GOE shared 100% sequence identity with “*Ca*. P. solani” 916/22 (2). GOE encodes the conserved metabolism and transport systems typical of phytoplasmas (16), but differs by encoding a riboflavin transport system. We identified homologs of virulence factors Stamp (17), Imp (18), Vmp-like proteins (19), and of the effector proteins SAP11, SAP54, and SAP05 (20, 21).

## Data Availability

Raw SMRT-reads have been deposited in the Sequence Read Archive under accession SRX24431439. A whole-genome project has been generated in GenBank under BioProject accession number PRJNA975436, and annotation is available under accession CP155828.
